# New Paradigms for the Study of Ocular Alphaherpesvirus Infections: Insights into the Use of Non-Traditional Host Model Systems

**DOI:** 10.3390/v9110349

**Published:** 2017-11-18

**Authors:** Matthew R. Pennington, Eric C. Ledbetter, Gerlinde R. Van de Walle

**Affiliations:** 1 Baker Institute for Animal Health, College of Veterinary Medicine, Cornell University, Ithaca, NY 14853, USA; mrp244@cornell.edu; 2 Department of Clinical Sciences, College of Veterinary Medicine, Cornell University, Ithaca, NY 14853, USA; ecl32@cornell.edu

**Keywords:** herpesvirus, HSV-1, CHV-1, FHV-1, ocular infection, model systems, natural host infection

## Abstract

Ocular herpesviruses, most notably human alphaherpesvirus 1 (HSV-1), canid alphaherpesvirus 1 (CHV-1) and felid alphaherpesvirus 1 (FHV-1), infect and cause severe disease that may lead to blindness. CHV-1 and FHV-1 have a pathogenesis and induce clinical disease in their hosts that is similar to HSV-1 ocular infections in humans, suggesting that infection of dogs and cats with CHV-1 and FHV-1, respectively, can be used as a comparative natural host model of herpesvirus-induced ocular disease. In this review, we discuss both strengths and limitations of the various available model systems to study ocular herpesvirus infection, with a focus on the use of these non-traditional virus-natural host models. Recent work has demonstrated the robustness and reproducibility of experimental ocular herpesvirus infections in dogs and cats, and, therefore, these non-traditional models can provide additional insights into the pathogenesis of ocular herpesvirus infections.

## 1. Introduction

Herpesviruses commonly infect and can cause ocular disease in a variety of species (Table 1). The prevalence of persistent infections that result in the presentation of ocular disease, however, is difficult to determine as it varies dramatically based on the population surveyed and the study methodology. Still, ocular diseases caused by human alphaherpesvirus 1 (HSV-1), canid alphaherpesvirus 1 (CHV-1) and felid alphaherpesvirus 1 (FHV-1) are among the most common causes of clinical herpesvirus-associated ocular disease and can lead to ocular pain, tissue destruction and blindness in severe cases [1,2,3]. The pathogenesis of these viruses is very similar and has been reviewed extensively [2,3,4,5,6]. Briefly, a person or animal becomes infected following contact with the ocular, nasal, or salivary secretions of an actively shedding host via the orofacial route. This leads to infection of epithelial cells of the oral mucosa, the cornea and/or the conjunctiva. In the case of FHV-1 and to a much lesser extent CHV-1, primary infection can also be established in the mucosae of the nasal septum, turbinate, nasopharynx, upper trachea, tonsils and mandibular lymph nodes, leading to the development of respiratory disease [5,7]. Following replication in epithelial cells, the virus is able to access the sensory neurons of the peripheral nervous system and travel via retrograde transport to the trigeminal ganglion (TG), where latency is established. Following reactivation, the virus can travel back down the axons and access all sites innervated by the TG, namely the oral cavity and the eye, where the virus will replicate again in epithelial cells of these tissues. This process can happen repeatedly and so tissue damage and clinical signs can be present during both primary infection and following reactivation, leading to recurrent ocular disease. Ocular disease develops as a result of the direct lysis of conjunctival or corneal epithelial cells. However, the most severe clinical signs are observed after the establishment of latency in the TG and subsequent cycles of reactivation. This leads to the formation of herpes stromal keratitis (HSK), which consists of severe corneal lesions, corneal neovascularization and infiltration of immune cells that contribute to destruction of the cornea, ultimately leading to blindness. Due to the striking similarities in pathogenesis of HSV-1, CHV-1 and FHV-1, studies in dogs and cats are proposed as valuable virus-natural host models to study the pathogenesis of human herpesvirus infections of the eye [2,5,8,9].

In this review, we discuss the strengths and limitations of different model systems available to study ocular herpesvirus infection, with an emphasis on the use of virus-natural host systems, the role of which has been generally underutilized in favor of studies in mice and rabbits [3,57]. The importance of studying infection in the natural host is well recognized for several viruses, particularly emerging zoonotic viruses, as there are often differences in the symptoms of the disease among the natural, transmission and human and/or animal hosts due to a variety of factors, including anatomical, physiological, metabolic, behavioral, genetic and immune [58]. Indeed, there is an emerging appreciation for (i) the recognition that in some cases, mouse models cannot fully recapitulate the disease presentation and progression as it occurs in the natural host and (ii) the value of studies in natural hosts as comparative models for human diseases. An excellent example is the infection of woodchucks with woodchuck hepatitis virus, which is well recognized as a comparative model of hepatitis B virus in humans due to the similarities in liver disease developed in both species following infection [59,60]. Since herpesviruses have been coevolving with their hosts over millions of years and are exquisitely adapted to their respective host [61,62,63], studies of ocular herpesvirus infection in the natural host are, therefore, valuable to better understand how different hosts respond to herpesvirus infection and/or which host factors are important for both establishment and control of infection. Moreover and importantly, virus-natural hosts models are useful to screen novel vaccines and antivirals based on better predictive outcomes.

## 2. In Vitro 2D Cell Culture Systems

### 2.1. Immortalized Cell Lines

A variety of immortalized cell lines have been used to study replication of herpesviruses capable of infecting the eye, as well as evaluating the efficacy of antiviral drugs. African green monkey kidney epithelial cells (Vero cells) are perhaps the most commonly used immortal cell line to study HSV-1 [64,65,66,67], although other cell types like CV-1, an African green monkey kidney fibroblast cell line, or Medical Research Council cell strain 5 (MRC-5), a human lung fibroblast cell line, are also frequently utilized [65,68]. For CHV-1 and FHV-1, Madin-Darby canine kidney cells (MDCK) and Crandell-Reese feline kidney (CRFK) cells, respectively, are almost exclusively used, with a major focus on evaluating antivirals in these cell lines [69,70,71,72,73]. The advantages of using immortalized cell lines are their ease of growing and the availability of cell lines from the respective host. However, it has to be noted that these cell lines are not derived from ocular tissues and, therefore, do not represent the cells that the virus encounters at the primary site of infection.

### 2.2. Primary Corneal Epithelial Cells

**Figure 1:**
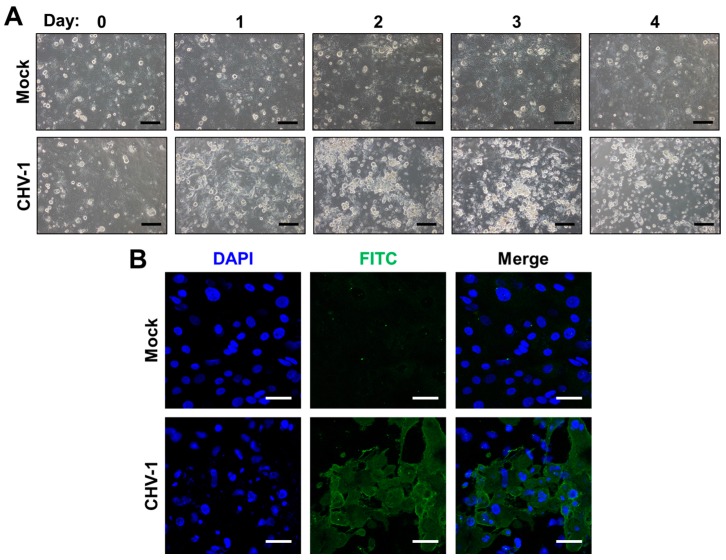
Canine corneal epithelial cells (CCECs) support the growth of canine herpesvirus type 1 CHV-1. CCECs were either mock-infected or infected at a multiplicity of infection (MOI) = 1 with the CHV-1 strain Duk. (**A**) Representative light micrographs of the course of the infection. Scale bar, 100 µm; (**B**) at 4 days post infection, CCECs were fixed in acetone and stained according to the manufacturer’s directions with a commercially available fluorescein isothiocynate (FITC)-conjugated anti-CHV-1 antibody (VMRD, Pullman, WA, USA; Cat#C5-F-CHV). Representative mock- and CHV-1-infected fields are shown. Scale bar, 100 µm. DAPI: 4’,6-diamidino-2-phenylindole.

Work in recent years has recognized that primary corneal epithelial cells (CECs), the primary ocular site of replication of HSV-1, FHV-1 and CHV-1, can be used to overcome some of the limitations of immortalized cells and have been successfully isolated from humans (HCECs), dogs (CCECs) and cats (FCECs) [74,75,76]. Isolation of primary corneal cells is accomplished either by outgrowth from a corneal explant, or via mechanical and enzymatic separation of corneal tissue. HCECs and FCECs have been described to support infection of HSV-1 and FHV-1, respectively [75,77,78]. Likewise, we have successfully isolated CCECs and confirmed that they can be infected with CHV-1 (Figure 1). FCECs and CCECs have the advantage in that they can be isolated from herpesvirus-free, healthy animals from commercial research breeding colonies (see Section 3.3). A disadvantage, however, is their limited lifespan. For example, FCECs are described to undergo senescence by passage 45, with gradual changes in morphology observable from passages 20–45 onwards [75]. In our experience, however, morphological transformation of FCECs to a fibroblastoid phenotype can occur as early as passage 10, quickly followed by extensive cell death in subsequent passages. One possibility to circumvent this is to immortalize CECs. Several such lines have been described, including the telomerase-immortalized human corneal epithelial cell line (hTCEpi), which expresses a human telomerase reverse transcriptase (hTERT) and has been shown to maintain normal karyotype and cell cycle kinetics to at least passage 240 [79] and a Simian virus 40 (SV40)-adenovirus vector transformed HCEC cell line that displayed a normal growth phenotype for more than 400 generations [80]. Many HCEC lines immortalized via telomerase, SV-40 and/or Rous sarcoma virus are commercially available via biological material resource centers such as American Type Culture Collection (ATCC).

### 2.3. Limitations of 2D Cell Culture Systems

One of the major limitations of 2D cell cultures is the absence of other cells and components of the ocular system, including the immune defense system. While 2D cell cultures can recapitulate the innate immune responses of epithelial cells to the virus, e.g. interferon production, they are unable to model more complex innate immune responses as well as the adaptive immune response that are the primary drivers of HSK [81]. Additionally, neovascularization, i.e. the ingrowth of blood vessels into the cornea, is a common component of HSK [82] and the breaking of corneal angiogenic privilege cannot be fully modeled in traditional 2D cell culture systems, though the growth patterns of endothelial cells can be analyzed individually in culture.

Additionally, it is well recognized that antiviral activity observed in 2D cell culture systems does not always translate into antiviral activity in vivo and potential drug toxicity is seldom accurately recapitulated in these simplified model systems [83]. Several examples of this exist in the search for effective antivirals against FHV-1. For instance, the nucleoside analogue idoxuridine was found to inhibit the replication of FHV-1 in CRFK at a half maximal effective concentration (EC_50_) ranging between 4.3–6.8 µM [69,84]. However, when evaluating its efficacy to treat cats presenting to the clinic with naturally acquired FHV-1-associated ocular disease, it was found that 4 out of 7 idoxuridine-treated FHV-1-infected cats exhibited no improvement or even worsened with therapy [85]. Another example is acyclovir, a nucleoside analogue that was shown to be effective at inhibiting FHV-1 replication in CRFK at EC_50_ values ranging between 57.9–66.6 µM [69,70]. When valacyclovir, a prodrug of acyclovir with better oral bioavailability, was evaluated in cats as a systemic therapy for FHV-1 ocular infections, it was found that all cats in the study develop acute conjunctivitis in addition to exhibiting systemic toxicity [86]. However, a study in which topical acyclovir treatment was evaluated in a limited number of client-owned cats did show some moderate improvement in clinical scores after a five time/day acyclovir treatment regimen for 21 days, which was initiated after a 21-day treatment with chlortetracycline due to suspected *Chlamydophila* spp. infection [87]. Thus, and despite initially promising results in CRFK, the use of acyclovir as a therapy for FHV-1 infection is still debated. On the other hand, the use of the nucleoside analogue cidofovir is an example where results in 2D cell culture did translate into efficacy of this drug in vivo. Cidofovir showed an EC_50_ of 11.0 µM in CRFK [69] and its antiviral activity and minimal toxicity was then confirmed in FCECs [88]. A follow-up study using topical cidofovir in experimentally-infected cats showed that this drug significantly reduced viral shedding and ocular clinical disease [89] and, consequently, cidofovir is currently one of the most commonly used topical antivirals used to treat ocular FHV-1 infection [90].

## 3. In Vitro 3D Cell Culture/Explant Systems

### 3.1. Corneal Facsimile

Corneal facsimiles, comprised of cornea cells grown in matrices, have sought to mimic the 3D structure of the cornea and cell-to-cell interactions in a renewable and cost-effective manner. One such model consists of human corneal stromal fibroblasts mixed with type I collagen in transwell plates overlaid with Matrigel to simulate the corneal stroma and basement membrane and this model has been used for corneal inflammation and adenovirus infection studies [91]. Subsequent improvements consisted of culturing epithelial cells over these stromal cells and the incorporation of an endothelium to create full thickness corneal equivalents [92]. Other models have been developed to recapitulate the 3D structure of specific portions of the cornea. For example, hydrogen scaffolds for corneal stromal tissues [93], decellularized human, porcine, or bovine corneas as a scaffold for reconstructing the corneal epithelium, anterior stroma and/or the endothelium [94,95,96] and hybrid electrospun poly (lactic-co-glycolide) mats combined with plastic compressed collagen matrices for entire corneas [97] have all been described as potential in vitro 3D models of the cornea. However and to our knowledge, none of these model systems have been used to study herpesvirus infections. Therefore, the use of these models to accurately recreate in vivo herpesvirus infection events remains to be determined.

### 3.2. Explants

**Figure 2:**
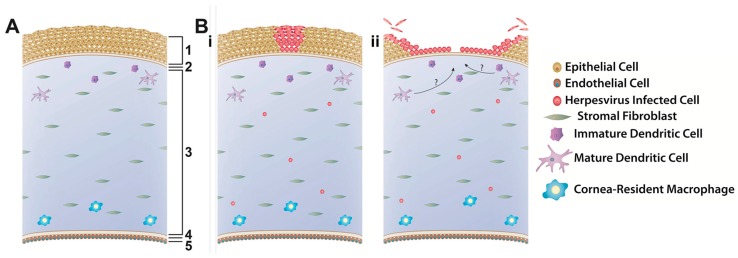
General progression of lytic herpesvirus infection in cornea explant models. (**A**) Structure of normal cornea with the five layers indicated: (1) epithelium, (2) Bowman’s layer (absent in felines and canines), (3) stroma, populated by stromal fibroblasts and various resident immune cells, (4) Descemet’s membrane and (5) endothelium; (**B**) following herpesvirus infection of the cornea explant, corneal epithelial cells become infected in a dose-dependent manner, resulting in the formation of plaques and dendrites. Unidentified stromal cells may also become infected (i). Following prolonged incubation, corneal epithelial cells are lysed and slough off into the culture media, leading to complete destruction of the epithelium (ii). The contribution of resident corneal immune cells in the host response to herpesvirus infection has not been formally investigated in explant cultures to date.

Explants—also referred to as ex vivo organ, organotypic, or organoid cultures—are highly sophisticated physiological systems that maintain the normal three-dimensional structures and cell-to-cell contacts of the tissue as they are found in vivo, without the disruption required to place such tissues into 2D cell culture. Moreover, since many pathological conditions, including ocular herpesvirus infection, involve more than one cell type, explants are considered to be much more physiologically relevant compared to cell culture systems [98]. Therefore, explants are proposed as stepping stones to bridge in vitro and in vivo models to validate cell culture results while limiting the amount of animal experimentation needed and thus satisfying the 3 R’s of animal research, i.e. replacement, reduction, and refinement [99]. Corneal or, more correctly, corneoscleral explants, were first developed as a means to prolong the time that human corneas could be preserved prior to transplantation [100] as well as to study wound healing in the human eye [101,102]. These explants were then expanded for the purpose of studying ocular herpesvirus infection. The methodology for obtaining and culturing these corneas is similar across species and consists of removing the eye from the donor and aseptically dissecting the corneoscleral buttons. The cornea is then placed epithelial side down and the endothelial cavity is filled with a 1% agarose solution to mimic the vitreous humor, providing support to maintain the normal 3D structure of the cornea [72]. Corneas may or may not be scarified prior to infection with herpesvirus and are either covered with media and cultured for up to 8 days, or placed in a rocking air-liquid interface and cultured for up to three weeks. Thus far, corneoscleral explants have been described to model HSV-1 infection using human, rabbit and pig corneas [103,104,105,106], CHV-1 infection using canine corneas [8] and FHV-1 infection using feline corneas [72,107]. The infection in these cornea models was shown to be similar across the different viruses and is depicted schematically in Figure 2. Briefly, corneal epithelial cells are the primary site of replication and viral plaques are formed upon cell lysis. The explants are typically infected at a high multiplicity of infection (MOI) to ensure infection and, as a result, virus is found uniformly across the epithelium, resulting in damage to the entire epithelium. However, using a porcine cornea explant to model HSV-1 growth, Thakkar et al. showed that dendritic ulcerations form in a virus inoculum-dependent manner, eventually leading to the formation of larger, geographic ulceration commonly observed in vivo [106]. Additionally, some models suggest that cells deeper in the corneal tissue, including endothelial cells and other unidentified cells, may also become infected [8,105] (Figure 2). Collectively, these data show that the infection patterns in corneal explants closely mimic the pathogenesis of ocular herpesvirus infections in vivo.

Human and rabbit corneal explants have been used to study the inhibition of herpes viral replication using phosphonoacetic acid (PAA) and to demonstrate the role of Checkpoint Kinase 2 (Chk2) in promoting HSV-1 replication in the cornea, suggesting that Chk2 inhibitors could be used to treat HSV-1 ocular infection [103,104]. Our group has used canine corneal explants to assess the local innate immune response and inflammation associated with CHV-1, similar to what has been done for HSV-1 infection of human corneal explants [8,105]. Feline corneal explants have been used by our group to study the efficacy and toxicity of acyclovir, cidofovir and the anti-human immunodeficiency virus (HIV) integrase inhibitor raltegravir against FHV-1 replication [72]. We found that the levels of efficacy of acyclovir and cidofovir in the explant model against FHV-1 were comparable to reported efficacy results in vivo (see Section 2.3), with cidofovir being significantly more effective than acyclovir, thus supporting the physiological relevance of this model. Furthermore, we showed that raltegravir is effective against FHV-1 using our feline corneal explant model and is non-toxic for corneal tissues, suggesting that topical raltegravir could prove to be a novel treatment option for FHV-1 ocular infection in cats [72].

Besides the corneal epithelial cells which play an important role as initiators of the innate immune response through a variety of pattern recognition receptors [108,109], the cornea also harbors a heterogeneous population of resident antigen-presenting cells in both stroma and epithelium [110,111] (Figure 2). These cells respond to cytokine cues from the epithelial cells and help to orchestrate the response of the systemic immune response that leads to clearance of the virus [112,113,114]. However, to our knowledge, no study has evaluated the presence, activity, or role in controlling the infection of these innate immune cells in corneal explants.

### 3.3. Limitations of 3D Cell Culture/Explant Systems

There are a couple of notable drawbacks using ex vivo cultures compared to 2D cell cultures, including higher costs, difficulty of access to donor tissue and a greater degree of variability between samples [103]. The source of the donor tissue could also be a concern. Corneas from human donors available for research are usually those deemed unsuitable for corneal transplantation and often come from older donors or donors with severe medical disorders [8,105]. Indeed, approximately 41% of donated human corneas are discarded from consideration for transplant, primarily because of medical contraindication or poor endothelial quality and thus become available for experimentation [115]. In contrast, canine and feline corneas can be collected from healthy research animals that are euthanized for unrelated reasons and are even commercially available as fresh grafts for transplantation purposes in these animals, resulting in a much better quality of these tissues.

Contamination with infectious agents is another limitation of using and culturing primary cornea tissues. For example, the presence of bacterial and fungal contamination has been studied in human corneas for transplantation. Overall, it was found that the contamination rate of cornea cultures ranges from 0.53 to 11% [116,117,118,119,120,121,122,123]. This contamination can be controlled to some extent by an initial decontamination procedure and subsequent culturing with penicillin, streptomycin, amphotericin B, voriconazole, or similar antibiotics and antimycotics. However, care must be taken to refresh the media periodically as the concentration of these agents can decline by as much as 86% during prolonged culture [124]. In addition and importantly, intra-corneal contamination with herpesviruses itself is a possibility. While HSK patients do have approximately 100 times more HSV-1 DNA in their corneas than healthy patients, HSV-1 is periodically shed in the tears of HSV-1 seropositive, yet asymptomatic individuals [125]. This has led to considerable debate in the field as to whether HSV-1 may establish latency in the cornea itself, though latency associated transcripts (LATs) have not been irrefutably detected in corneal tissues, or whether the shedding is a result of frequent subclinical replication in the cornea [126,127]. Nevertheless, studies have shown that between 1.8 to 38% of donor corneas for transplantation were contaminated with HSV-1 DNA using PCR [128,129,130,131,132]. One study further demonstrated that infectious HSV-1 could be isolated successfully from 7 PCR positive cases, although it was noted that these donors had a history of long-standing or severe illnesses, often in the hospital, that may have contributed to this active viral shedding [133]. Likewise, FHV-1 has been detected in the corneas of clinically health cats at a rate of 20% [134] and although the frequency of CHV-1 DNA in clinically healthy dog corneas has not been evaluated to our knowledge, it is most likely similar to FHV-1. It is currently not clear how the presence of herpesviral DNA or even potentially low levels of infectious virus impact experimental outcomes in corneal explants. However, the likelihood of herpesvirus contamination is likely to be low. Furthermore, specific pathogen-free (SPF)—including herpesvirus-free—dogs and cats are available commercially from laboratory animal supply companies and so the use of corneas from these animals could, therefore, address the concern of low-level herpesvirus infection of donor corneas. If such animals are not available or human tissue is to be used, negative controls of uninfected matched corneas from the same donor should ideally be included.

## 4. In Vivo Systems

Rabbits and mice are commonly used to study HSV-1 ocular infection and this has been reviewed extensively [3,57]. Here, we will give a brief overview of the general pathogenesis of ocular HSV-1 in these traditional models and we will elaborate more on the non-traditional model species such as dogs and cats. The latter two models represent virus-natural host infection models and as such, can complement studies on the pathogenesis of human herpesvirus infections of the eye.

### 4.1. Mice

Studies in mice constitute the majority of our understanding on HSV-1 pathogenesis in vivo, including the immune response and latency. Mice are more commonly used than rabbits based on their small size, which reduces the amount of drugs and chemicals required for testing, the cost of boarding and the ready availability of inbred strains with the same genetic makeup [3]. In addition, a large number of knockout and transgenic mice exist that allows for a detailed dissection of pathways and factors involved in ocular herpesvirus infections [57]. C57BL/6 strains are most commonly used and are infected in one or both eyes by scarifying the cornea using a needle to induce physical disruption of the corneal epithelial cells to facilitate infection. These mice are then infected with HSV-1 at titers ranging between 10^3^ and 2 × 10^6^ plaque forming units (PFU) per eye. The HSV-1 McKrae strain is most commonly used for animal infections, in part due to the ability of this virus strain to establish higher genome numbers per neuron [135]. In addition, the RE and 17Syn+ HSV-1 strains are also frequently used, both of which establish high numbers of genomes per neuron when compared to other virus strains such as KOS [136,137,138,139,140]. These virus- and mouse strain-dependent responses could help to further elucidate virus and host factors involved in the development of recurrent ocular herpesvirus-associated keratitis.

Despite the many strengths of using mice, as described above, there are a few limitations. The small size of the cornea makes it difficult to assess corneal lesions and there is a corresponding limited amount of tear film volume making the assessment of viral shedding more challenging. Furthermore, the small size of harvested tissue can potentially limit subsequent analysis. The major limitation of the mouse model, however, is that it is not the ideal model for studying viral reactivation. Indeed, there is controversy as to whether spontaneous reactivation in immunocompetent mice actually occurs. Therefore, reactivation is typically induced by either raising the body temperature of the animal to high levels of 42 °C or by directly exposing the cornea to ultraviolet light, among other methods [57,141,142,143]. Even with these methods, only low levels of induced reactivation are observed in mice, albeit with a higher efficiency in the BALB/c compared to C57BL/6 mice [3,57].

### 4.2. Rabbits

Rabbits are also used to study HSV-1 ocular infection and they address some of the limitations of the mouse model. Their larger corneal surface can be imaged more easily and quantified by slit-lamp examination. There is also a higher amount of corneal tissue and tear film available for downstream analyses. New Zealand white rabbits are almost exclusively used as their non-pigmented eyes allow for easier examination, though Dutch Belted rabbits are also used due to their smaller size and subsequently cheaper housing and treatment costs. Less variation in experimental design is also reported in studies using the rabbit model compared to the mouse model. The McKrae strain is typically used to infect the non-scarified eye at a concentration of 2 × 10^5^ PFU/eye, due to this strain’s high reactivation frequency [144,145,146]. However, other strains of HSV-1 (Rodanus, RE, F, KOS, 17Syn+ and E-43) can also spontaneous reactivate and are, therefore, occasionally used [57,147].

Despite the strength of the rabbit model regarding this spontaneous reactivation, it does have a number of limitations that contribute to its reduced use compared to the mouse model. Inbred rabbit strains are expensive and can be difficult to obtain. Also, fewer transgenic strains exist compared to mice, although some have been used to study ocular herpesvirus infection, such as the humanized human leukocyte antigen (HLA)-A*0201 transgenic rabbit, which has been used to evaluate the efficacy of a CD8+ T cell epitope-based prophylactic vaccine against ocular HSV-1 infection [148].

### 4.3. Dogs

**Figure 3:**
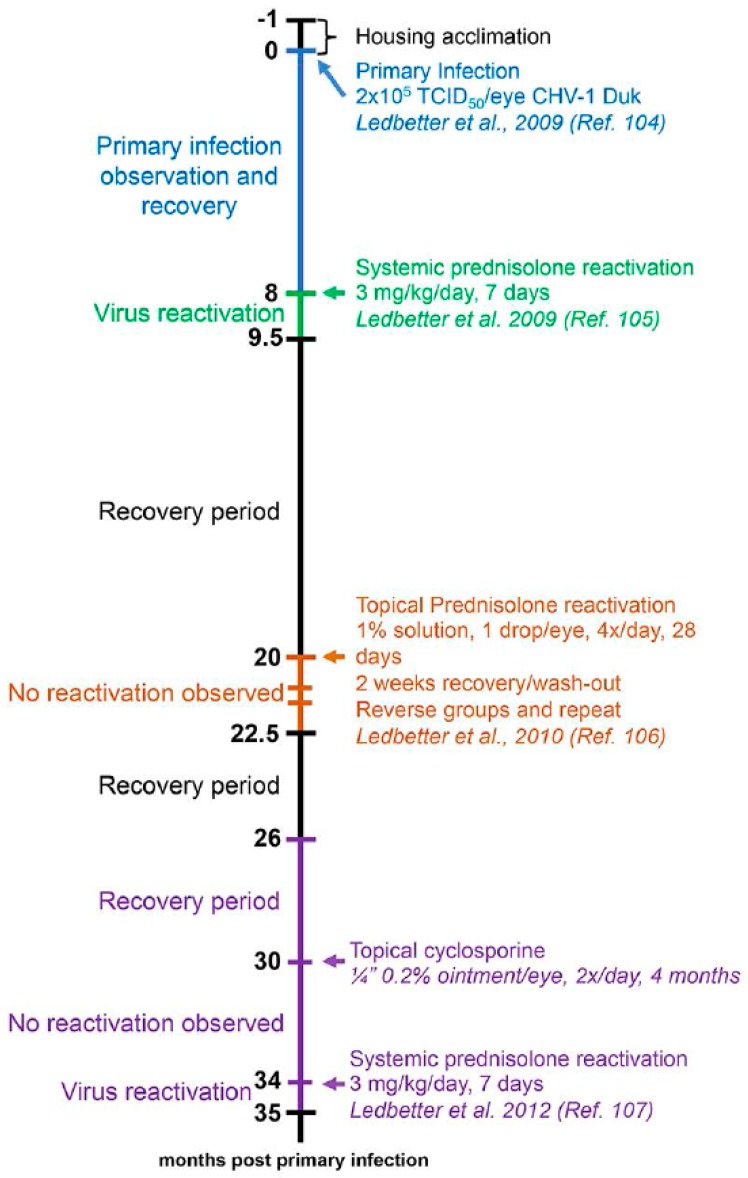
Timeline of the development and validation of the recurrent experimental ocular CHV-1 infection model in dogs. Dogs were initially infected with 2 × 10^5^ tissue culture infectious dose 50 (TCID_50_)/eye CHV-1 Duk strain. Virus reactivation was then attempted using different methods in the same colonies of dogs at various times post infection. Different infection or reactivation studies are indicated with different colors.

**Figure 4:**
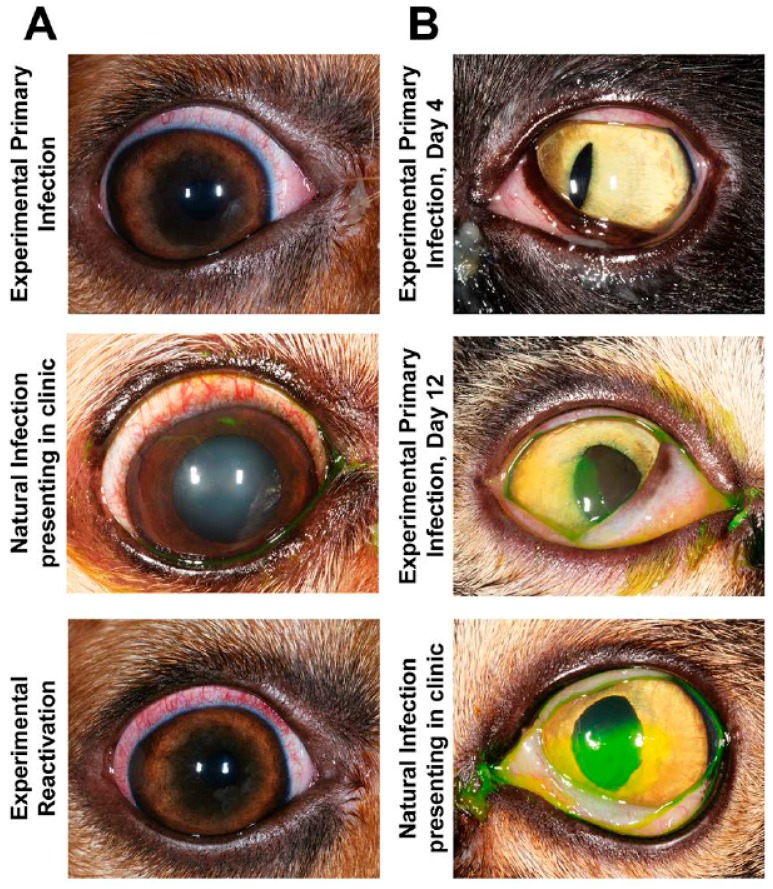
Clinical presentation of experimental and clinical infection of ocular herpesviruses in dogs and cats. (**A**) Representative CHV-1-associated ocular disease in dogs at day 10 post primary experimental infection, natural infection presenting to the clinic, or at day 7 post reactivation with systemic prednisolone. Experimentally infected dog eyes were stained with lissamine green, although no retention in either dog was observed. Naturally infected dog eyes were stained with fluorescein dye to visualize corneal ulcers; (**B**) representative feline herpesvirus type 1 FHV-1-associated ocular disease in cats following primary experimental infection at day 4 post infection, with conjunctivitis only and at day 12 post infection, with both corneal ulceration and conjunctivitis, or natural infection presenting to the clinic with both corneal ulceration and conjunctivitis. Cat eyes were stained with fluorescein dye to visualize corneal ulcers.

Ocular CHV-1 infection in dogs is a good representation of ocular infections in humans due to similar pathogenesis and clinical presentation of the disease [2,149]. Whereas prior work focused on the fatal hemorrhagic form of CHV-1 by infecting newborn puppies, Wright and Cornwell described in 1969 the experimental infections of six-week old puppies with 10^5^ tissue culture infectious dose 50 (TCID_50_) of a CHV-1 field strain via different routes, including the intraconjunctival route. They found that one out of the five puppies infected via this route developed ocular discharge from the infected eye at day 3 post infection (pi), shed virus and showed infiltration of lymphocytes and macrophages into the infected conjunctiva as well as necrosis of the epithelial cells [150]. Decades later, Ledbetter et al. developed and optimized a CHV-1 reactivation model using a single colony of experimentally infected dogs [151] and a schematic timeline for these studies is shown in Figure 3. Briefly, the right eyes of 18-month old SPF beagles were infected with 2 × 10^5^ TCID of CHV-1-Duk, a CHV-1 field strain isolated from a dog presenting with dendritic ulcerative keratitis, either without scarification or using the microtrephination technique, a mild form of scarification (Figure 3, blue color). This resulted in all 8 CHV-1-infected dogs shedding CHV-1, with viral loads peaking at 5 days pi and then steadily declining until no virus could be recovered from ocular swabs by 15 days pi. Clinical scores peaked at approximately 7–10 days pi, after which they gradually declined until clinical signs were no longer present, around day 30 pi [151]. No spontaneous reactivation was observed in these dogs until the end of the study, which was 8 months pi (or day 224 pi). A mild to moderate conjunctivitis, characterized by intermittent blepharospasm, conjunctival hyperemia, chemosis and mucoid to mucopurulent ocular discharge, was typically observed in these experimentally infected dogs during primary infection (Figure 4A). The development of corneal ulcerations, however, was not as frequent in experimentally infected dogs when compared to natural CHV-1 infections, where this is a common clinical sign (Figure 4A).

Following the 8-month recovery period, reactivation was induced in six dogs from the aforementioned primary infection study (Figure 3, green color), confirmed to be clinically healthy and not shedding virus, with 3.0 mg/kg of the immunosuppressant prednisolone given orally every day for 7 days. Peak viral shedding was observed at 10 days post reactivation and 5 out of the 6 dogs developed bilateral conjunctivitis or ulcerative keratitis (only 1 dog developed corneal ulcers) between 3 and 18 days post reactivation [152]. This observed clinical disease presentation was similar to the primary experimental infection (Figure 4A). Following a second 10-month recovery period, these animals were used to evaluate the ability of topical prednisolone to induce reactivation when delivered as a 1% ophthalmic solution with 1 drop per eye four times per day for 28 days (Figure 3, orange color). Following a 2-week steroid wash-out period, the groups were reversed and the treatments were repeated. This treatment, however, did not result in virus shedding and clinical disease beyond some mild conjunctivitis in some dogs that was not believed to be virus-related [153]. Six months later, or 26 months following primary infection, the same animals were given a topical treatment with the immunosuppressant cyclosporine (a one-quarter inch strip of 0.2% ointment, twice daily in both eyes) and, likewise, no reactivation was observed [154] (Figure 3, purple color). It should be noted, however, that reactivation could be induced again in these animals when subjected to systemic prednisolone treatment at 33–36 weeks following cyclosporine treatment [154] (Figure 3, purple color). An additional study in a separate colony of dogs indicated that the immunosuppressant cyclophosphamide (200 mg/m^2^ intravenous) could not induce reactivation, however, systemic prednisolone was again used to successfully induce reactivation in these animals at 6 months post cyclophosphamide treatment [155]. These results indicate that colonies of latently infected dogs can be maintained and used for both primary infection as well as repeated reactivation studies. Finally, an additional study demonstrated that strontium-90 β radiotherapy (36.7 cGy/s), which is frequently used as an adjuvant treatment for a variety of ocular surface, adnexal neoplasms and inflammatory conditions in dogs, was not capable of inducing reactivation, [156], indicating that this therapy is associated with a low risk of recurrent ocular herpes disease.

The systemic prednisolone reactivation model was also used to evaluate the abilities of topical cidofovir, trifluridine and ganciclovir to either control or prevent CHV-1-associated ocular disease, as well as to evaluate the efficacy of a subunit vaccine [71,73,157,158]. The experimental design of these studies was similar and consisted of a minimum 12-week acclimation period following acquisition of the dogs, infecting the dogs as described above, a 12-month recovery period to allow for the establishment of latent infection and then reactivation using oral prednisolone for 7 days beginning on study day 1. All dogs reactivated successfully using systemic prednisolone therapy, with virus shedding peaking around day 9 post reactivation and clinical scores peaking on days 7–10 post reactivation. Topical antiviral therapy was initiated at various days for different lengths of time. Cidofovir was found to be effective at reducing viral shedding but was associated with increased conjunctival and corneal leukocyte infiltration as quantified with in vivo confocal microscopy and exacerbation of ocular disease as detected by clinical ophthalmic examination [71]. In contrast, trifluridine was highly effective to control the course of clinical disease and reduce viral shedding [157]. For the subunit vaccine study, dogs were vaccinated twice at 57 and 15 days prior to the administration of oral prednisolone to latently infected dogs. The vaccinations did not prevent the development of ocular disease or viral shedding. However, they did reduce the clinical ocular disease scores in the postvaccinal period short-term and increased CHV-1 specific immunity long-term [158].

Collectively, the data from the experimental ocular CHV-1 dog model showed that (i) CHV-1 can be readily and reproducibly reactivated in CHV-1 latently infected animals using systemic prednisolone with a high rate of reactivation, demonstrating the robustness of this model; and (ii) CHV-1 either does not reactivate spontaneously in immunocompetent adult dogs or reactivation occurs at a level below the detection limit of the assays utilized in these studies.

### 4.4. Cats

Ocular herpesvirus infections in cats is also proposed to be a good representation of ocular infections in humans due to similar pathogenesis and clinical presentation of the disease [5]. Experimental ocular FHV-1 infection models in cats have been used primarily to study the efficacy of antiviral therapies. One of the first experimental in vivo studies used SPF cats that were infected with 10^5^ PFU per eye of different FHV-1 strains, followed by scarification of the cornea. With this approach, conjunctival epithelial cell infection was apparent by day 4 pi and corneal epithelium infection by day 8 pi [159]. Other experimental models applied between 1.5 and 3 × 10^6^ PFU per eye of various field strains to the non-scarified eye and this reliably resulted in peak viral titers at approximately 3 days pi and ocular clinical scores around day 7–8 pi, after which they declined over the following 14–21 days [86,89,160,161]. Experimental primary FHV-1 infection is characterized by the development of conjunctivitis, dendritic or geographic ulcerative keratitis and blepharitis (Figure 4B). Cats may also exhibit varying degrees of blepharospasm, conjunctival hyperemia, chemosis and ocular discharge, which may appear serious or mucopurulent. Typically, no major differences in the clinical signs of experimentally versus naturally infected cats are observed (Figure 4B).

In contrast to HSV-1 infection studies in mice and rabbits, the FHV-1 strain used to inoculate cats is not of critical importance. Evidence for that was first provided by the study of Nasisse et al. in which they observed no difference in the presentation and course of the disease following infection with five different strains of FHV-1, including 4 field strains [159]. Moreover, and in general, FHV-1 is accepted to have little strain variation based on the observations that all strains belong to one serotype antigenically and isolates are relatively homogeneous by restriction enzyme digestion [4,162]. Finally, it was recently shown that the genomes of 24 clinical strains of FHV-1, collected over a period of 40 years, showed remarkably low levels of diversity and no potential genetic determinants of virulence could be identified [163].

In addition to the acute models of ocular FHV-1 infection, a preliminary latency-reactivation model has also been described [164]. For this, 14 SPF cats were infected with 7 × 10^4^ PFU of an unspecified plaque-purified field strain of FHV-1. All cats showed clinical signs consistent with primary FHV-1 infection, recovered without treatment and no indication of disease was observed in the 5-month follow-up period. Reactivation of FHV-1 was induced at 5.5 months pi, using a single dose of methylprednisolone acetate (5 mg/kg) intramuscularly and 3 out of 14 cats (21%) developed bilateral conjunctivitis. However, the authors noted that 12 out of 14 cats (86%) had FHV-1 detectable in ocular swabs by PCR just prior to corticosteroid administration. Since they had recently rehoused the cats from group housing to individual cages in order to conduct the reactivation study, it was hypothesized that this event induced the high rate of virus shedding. While no clinical disease was noted prior to methylprednisolone injection, viral shedding before rehousing was not determined. Therefore, while corticosteroid injection did appear to induce clinical disease in a small percentage of cats, it is not clear whether the reactivation was due to the treatment or the rehousing event. Additionally, it is possible that viral shedding was not controlled after primary infection, although the cats recovered from clinical disease during the 5-month recovery period. Still, this study illustrates that reactivation can potentially be induced in cats using immunosuppression similar to what has been demonstrated for the dog model, although additional studies are needed to further validate this reactivation model.

### 4.5. Limitations of Non-Traditional In Vivo Models

Despite the unique and important strength of fully recapitulating the pathogenesis of ocular herpesvirus infections in a natural host setting, the use of cats and dogs as comparative models for HSV-1 infection has certain limitations. Cats and dogs are much more expensive to buy and maintain compared to mice and rabbits and specific housing is required. Additionally, there are important ethical and public-perception considerations associated with the use of companion animals as experimental animal models [165,166]. Moreover, no transgenic cats and dogs exist yet that could be useful for ocular herpesvirus infection studies, although transgenic red fluorescent protein-expressing cats and dogs have been described [167,168]. The development of transgenic cats and dogs to assist in the study or treatment of various diseases requires successful in vitro fertilization approaches, which have been described in cats since the early 1990s and in dogs only recently [169,170].

**Figure 5:**
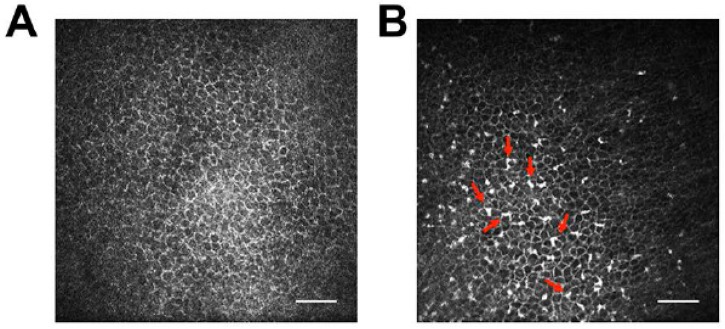
In vivo confocal microscopy of canine corneas. Representative confocal photomicrographs of the cornea of an uninfected dog (**A**) and following ocular CHV-1 infection at day 10 post infection (**B**). Note the corneal infiltration with leukocytes following virus infection, which appear as highly reflective, irregularly shaped white cells within the corneal epithelium (see arrows). In vivo confocal microscopy of cats yields similar photomicrographs. Scale bar, 50 µm.

Also and in contrast to rodent models, molecular tools to study the hosts’ immune reactions in response to FHV-1 and CHV-1 infections are largely lacking, although several groups are actively working on expanding this tool kit for virology and other studies. For example, a TaqMan-based qPCR assay targeting 12 genes has been described to study the innate immune response of feline embryonic fibroblasts to infection with feline leukemia virus in culture [171] and our group has utilized a canine microarray to evaluate the local immune response in response to ocular CHV-1 infection in the air-liquid canine corneal organ culture model [8]. Furthermore, as both the dog and cat genomes have been sequenced and at least partially annotated, transcriptome profiling is possible. RNA sequencing has been used to assess the transcriptome profiles of canine-derived tumors [172,173] and to study the immunological response of canine dermal fibroblasts to burdock extract treatment [174], among other studies. However, to our knowledge, no study has used RNA sequencing of virus-infected canine cells. In contrast, RNA sequencing has been used to profile virus infection of feline cells. For example, it has been used to assess the transcriptome profile of feline immunodeficiency virus (FIV) infected feline T-lymphocytes [175] and we recently performed RNA sequencing of FHV-1-infected FCECs. In addition, many antibody companies are expanding their repertoires beyond mouse and human and new antibodies that are either specific for or cross-react with canine and feline immune cell epitopes are being developed. Furthermore and a possible way to circumvent the lack of antibodies against immune cells is to use in vivo confocal microscopic examination, which is routinely used in ophthalmology clinics to assess leukocyte infiltrations in real time in the cornea and conjunctiva of dogs (Figure 5) and cats. For example, in vivo confocal microscopy was used to demonstrate that topical cidofovir treatment induced increased leukocyte infiltration into the cornea and conjunctiva of CHV-1-infected dogs, an undesired side effect that contributed to the ocular toxicity of this drug [71]. In vivo confocal microscopy has been used to define the structure of the normal cat cornea [176,177] and was recently used by our group to assess leukocyte infiltrates in cats with experimental ocular FHV-1 infection.

## 5. Conclusions and Future Prospects

Various models to study ocular herpesviruses exist, ranging from 2D in vitro cell culture over 3D ex vivo organ cultures to in vivo experimental eye infection models. While mice and rabbits have been used traditionally for in vivo studies using HSV-1, recent work with CHV-1 and FHV-1 in dogs and cats, respectively, has shown the robustness and reproducibility of these virus-natural host models to study ocular herpesvirus infections and disease. Many studies have shown that a successful translation of research from in vitro cell culture to in vivo experiments and from mouse or rabbit work to human applications, is not always guaranteed. Therefore, the choice of an appropriate model system is of the utmost importance. An ideal model system should (i) be a natural host for the given virus [5,178]; (ii) recapitulate the tissues of relevant importance with the appropriate cytoarchitecture [179]; (iii) recreate the characteristics of the disease, including relevant immune responses [114,180]; and (iv) be renewable or reusable [99]. The importance of each criterion varies based on the experiment at hand. Despite the limitations in working with non-traditional animal models, either as explants or in vivo, we believe in their value to complement results obtained with the existing traditional models, especially with an expansion of the tools to study these species and their increasing acceptance in the research field. Indeed, dogs and cats, amongst other domesticated veterinary species, are naturally infected with various pathogens that are closely related to pathogens that infect humans, making these animals a translational valuable model for viral pathogenesis studies. Moreover, veterinary viruses are frequently used as surrogates of human viruses in the discovery and development of novel antiviral drugs and vaccines against human pathogens and, as highlighted in this review, these types of studies are also applicable for ocular herpesviruses. Such studies may provide new insights into how different hosts respond to infection and/or which host factors are important for the establishment and control of infection, ultimately leading to the benefit of both human and animal health.

## Figures and Tables

**Table 1:** Overview of herpesviruses reported in literature to frequently cause ocular disease.

Virus	Abbreviations	Subfamily	Associated Ocular Diseases	Overall Prevalence	Herpesvirus-Associated Ocular Disease Prevalence	References
Human alphaherpesvirus 1	HHV-1/HSV-1	Simplexvirus	Corneal lesions, stromal & epithelial keratitis, conjunctivitis	67–90%	12–36/100,000	[3,10,11,12,13,14]
Canid alphaherpesvirus 1	CHV-1	Varicellovirus	Corneal lesions, stromal & epithelial keratitis, conjunctivitis	21–98%	Unknown	[2,15,16,17,18,19,20]
Felid alphaherpesvirus 1	FHV-1	Varicellovirus	Corneal lesions, stromal & epithelial keratitis, conjunctivitis	40–97%	Unknown	[21,22,23,24,25]
Human alphaherpesvirus 3	HHV-3/VZV	Varicellovirus	Herpes zoster ophthalmicus	＞ 95%	19–31/100,000	[26,27,28]
Equid alphaherpesvirus 1	EHV-1	Varicellovirus	Chorioretinitis	52%-“endemic”	50–90% of choroidal lesions in experimental infection	[29,30,31,32]
Equid gammaherpesvirus 2	EHV-2	Percavirus	Keratoconjunctivitis	51–93%	8–60% of keratoconjunctivitis cases tested	[33,34,35,36,37,38]
Bovine alphaherpesvirus 1	BoHV-1	Varicellovirus	Keratoconjunctivitis	20–97%	4.95/100	[39,40,41]
Bovine gammaherpesvirus 4	BoHV-4	Rhadinovirus	Keratoconjunctivitis & ocular discharge	21–35%	Unknown	[42,43,44,45]
Alcelpahine gammaherpesvirus 1 & Ovine gammaherpesvirus 2	AlHV-1 OvHV-2	Macavirus	Ocular discharge	29–77%	Typical symptom of malignant catarrhal fever	[46,47,48,49]
Cervid alphaherpesvirus 1 & 2	CvHV-1 CvHV-2	Varicellovirus	Keratoconjunctivitis & keratitis	18–47%	~5% in free-ranging, 30% in animals	[50,51,52,53,54,55]
Otariid herpesviruses & Phocid herpesviruses (Various species)	OtHV PhHV	Gammaherpes-viruses	Corneal lesions, keratoconjunctivitis	26–76%	Unknown	[56]
